# Retrovirus Drugs-Loaded PEGylated PAMAM for Prolonging Drug Release and Enhancing Efficiency in HIV Treatment

**DOI:** 10.3390/polym14010114

**Published:** 2021-12-29

**Authors:** Thi Thinh Nguyen, Bao Phu Nguyen, Dinh Tien Dung Nguyen, Ngoc Hoi Nguyen, Dai Hai Nguyen, Cuu Khoa Nguyen

**Affiliations:** 1 Institute of Drug Quality Control, Ho Chi Minh City 70000, Vietnam; nguyenthithinh73@gmail.com; 2 Graduate University of Science and Technology, Vietnam Academy of Science and Technology, Hanoi 10000, Vietnam; hoi83bmt@gmail.com (N.H.N.); nguyendaihai0511@gmail.com (D.H.N.); 3 Faculty of Environment and Natural Resources, Ho Chi Minh City University of Technology, Ho Chi Minh City 70000, Vietnam; nguyenphubao1969@gmail.com; 4 Institute of Fundamental and Applied Sciences, Duy Tan University, Ho Chi Minh City 70000, Vietnam; nguyendtiendung@duytan.edu.vn; 5 Faculty of Natural Science, Duy Tan University, Danang City 550000, Vietnam; 6 Institute of Applied Materials Science, Vietnam Academy of Science and Technology, Ho Chi Minh City 70000, Vietnam

**Keywords:** antiretroviral, HIV/AIDS, pamam dendrimers, pegylated, prolong release, side effects

## Abstract

Polyamidoamine dendrimer (PAMAM) with its unique characteristics emerges as a potential drug delivery system which can prolong releasing time, reduce the side effects but still retaining treatment efficiency. In this study, methoxy polyethylene glycol modified PAMAM generation 3.0 (G3.0@mPEG) is prepared and characterized via ^1^H-NMR, FT-IR, and TEM. Subsequently, two antiretroviral agents (ARV) including lamivudine (3TC) and zidovudine (AZT) are individually encapsulated into G3.0@mPEG. The drug-loading efficiency, drug release profile, cytotoxicity and anti-HIV activity are then evaluated. The results illustrate that G3.0@mPEG particles are spherical with a size of 34.5 ± 0.2 nm and a drug loading content of about 9%. Both G3.0@mPEG and ARV@G3.0@mPEG show no cytotoxicity on BJ cells, and G3.0@mPEG loading 3TC and AZT performs sustained drug release behavior which is best fitted with the Korsmeyer–Peppas model. Finally, the anti-HIV activity of ARV via Enzymatic Assay of Pepsin is retained after being loaded into the G3.0@mPEG, in which about 36% of pepsin activity was inhibited by AZT at the concentration of 0.226 mM. Overall, PAMAM G3.0@mPEG is a promising nanocarrier system for loading ARV in HIV treatment and prevention.

## 1. Introduction

Retroviruses (family Retroviridae) have been receiving a lot of attention due to their association with several serious illness such as cancers, acquired immunodeficiency syndrome (AIDS) and neurologic diseases. In term of mechanism, retroviruses contain a diploid RNA genome as their genetic material and convert their RNA genome to double-stranded DNA after infecting the host cells. This dual genetic system allows retroviruses to transmit from cell to cell as packaged RNA but still leave a DNA copy in the infected cell to transmit from one cell generation to the next [1]. Being first diagnosed in 1981 and classified as complex retroviruses, the human immunodeficiency viruses (HIV) infect humans and cause AIDS—one of the world’s most serious public health challenges [2]. According to UNAIDS, 79.3 million people have become infected with HIV and 36.3 million people have died from AIDS-related illnesses since the start of the epidemic [3]. To prevent the HIV/AIDS epidemic, antiretroviral drugs (ARV) including Lamivudine (3TC) and zidovudine (AZT) that work by inhibiting reverse transcription and disrupting the synthesis of DNA from viral RNA have been used [4,5,6]. Although the current ARV partially restores the immune system and significantly increases the life quality of infected people, these drugs in free forms are highly toxic, causing many serious side effects such as acute liver damage and metabolic disorders [7,8]. In addition, difficulties in ARV assessment, low oral bioavailability due to poor aqueous solubility, development of multi-drug resistant strains, low drug accumulation at viral reservoirs and poor adherence also threaten the HIV preventing efforts. Therefore, it is meaningful to find methods that can reduce the adverse reactions and enhance the treatment effects as well as prolong the acting of ARV.

Recently, nanotechnology strategies have been applied and showed their potentials in HIV prevention and treatment [9,10,11]. With nanotechnology solution, ARV are encapsulated into nanocarrier systems to prolong drug-releasing time and reduce the side effects while ensuring the treatment efficiency of the drugs [12,13]. Among the nanocarrier systems polyamidoamine (PAMAM) dendrimers has emerged as a potential candidate for drug delivery applications [14,15]. With the characteristics of monodisperse, high aqueous solubility, a polyvalent surface and dendritic architecture, PAMAM dendrimers are similar to biological systems, and could therefore augment the ability of penetration and functional performance [16,17]. Moreover, thanks to the architecture of PAMAM dendrimers, their hydrophobic pockets inside could enlarge the capacity of loading, increase the solubility as well as decrease the side effects of ARV molecules. Nevertheless, the unique hyper branched structure of PAMAM dendrites is expected to decelerate the release rate of drugs, contributing to prolong the release of ARV. However, higher-generation dendrimers and cationic dendrimers showed higher cytotoxicity [18]. Luckily, this drawback could be overcome by the modification of PAMAM’s surface with polyethylene glycol (PEG) [19,20,21]. This helps not only reduce the PAMAM’s cytotoxicity but also enhance the encapsulate capacity, permeability, and blood residence time of the modified particles [22]. Overall, PEGylated PAMAM is a potential carrier for encapsulating retrovirus drugs applied in HIV treatment.

In this study, the PEGylated PAMAM generation 3.0 nanocarrier encapsulating ARV (ARV@G3.0@mPEG) was synthesized with the aim to prolong drug release and enhance efficiency of ARV in HIV treatment. Firstly, G3.0@mPEG was synthesized and characterized by ^1^H Proton nuclear magnetic resonance (^1^H-NMR), Fourier-transform infrared spectroscopy (FT-IR) and Transmission electron microscopes (TEM). Then, two types of ARV including 3TC and AZT were individually loaded into G3.0@mPEG. The drug-loaded efficiency and prolonged drug-released ability of the system were evaluated. Importantly, the cytotoxicity of the obtained G3.0@mPEG and ARV@G3.0@mPEG was tested via MTT assay on human fibroblast BJ cell line; meanwhile the anti-HIV evaluation was conducted with pepsin as an alternative target enzyme for HIV-1 protease [23,24]. This study could contribute promising findings in the application of PAMAM G3.0@mPEG loading ARV in HIV treatment and prevention. 

## 2. Materials and Methods

### 2.1. Materials

Methoxy polyethylene glycol (mPEG, molecular weight = 5000 Da) and 4-nitrophenyl chloroformate (NPC) were purchased from Sigma Aldrich (St. Louis, MO, USA). Ethylene diamine (EDA), methyl acrylate (MA) and pepsin (PS) were obtained from Merck (Darmstadt, Germany). Methanol and tetrahydrofuran (THF) were purchased from Fisher Scientific (NJ, USA). Dialysis membrane with molecular weight cut-off (MWCO) of 3.5 kDa was bought from Spectra/Por^®^ (Texas City, TX, USA). Lamivudine 99.5% (3TC, LOT:R088C0) and zidovudine 99.6% (AZT, LOT:R052L0) were U.S.P grade. 

For cell culture, Dulbecco’s modified Eagle’s medium (DMEM), penicillin-streptomycin solution (10,000 U/mL), fetal bovine serum (FBS) were purchased from Sigma-Aldrich (Singapore). Other chemicals and solvents were used without further purification.

### 2.2. Synthesis of PAMAM G3.0

**Scheme 1:**
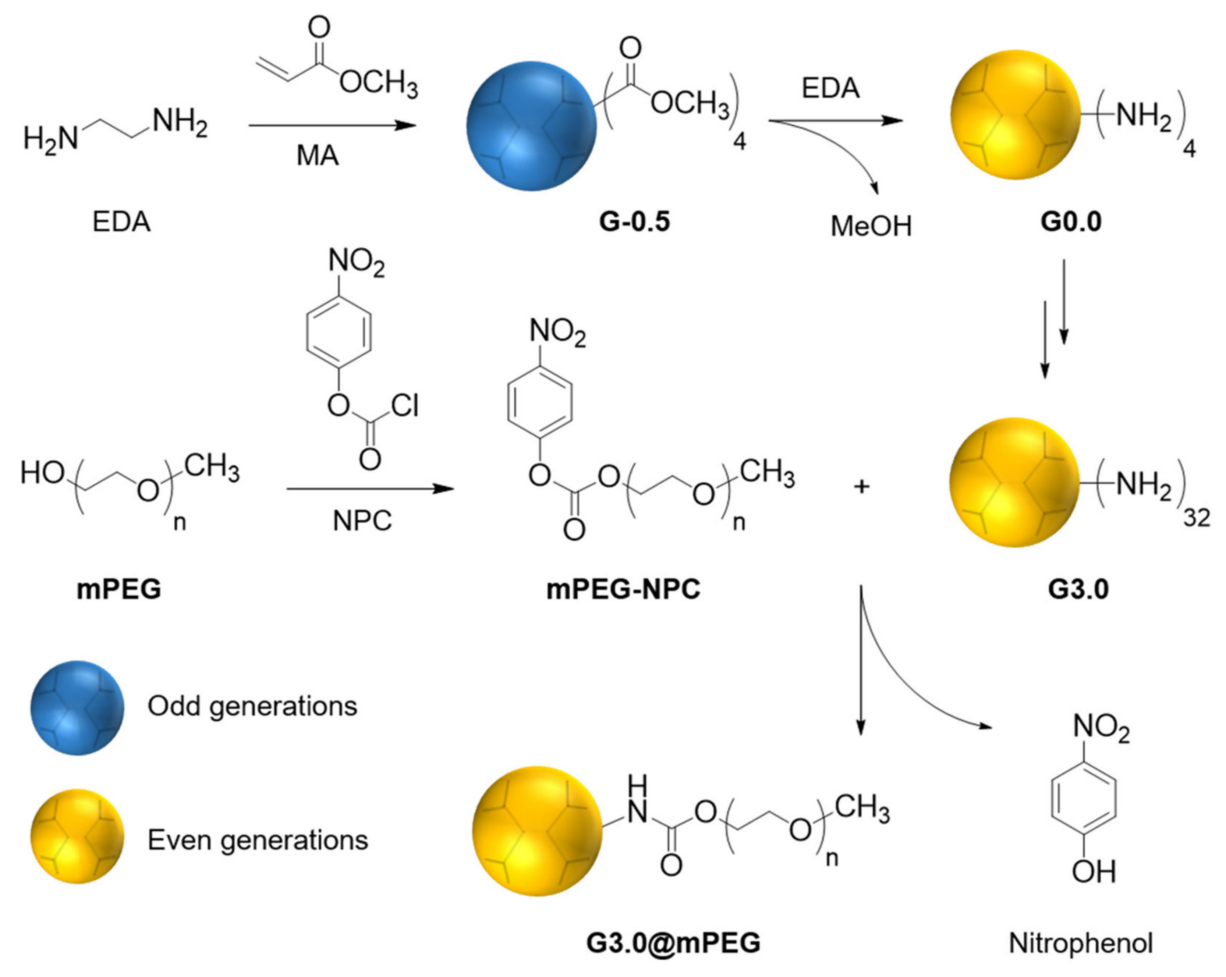
Schematic representation of the formation of PAMAM G3.0 and PAMAM G3.0@mPEG.

G3.0 was synthesized, generation by generation, via Michael reaction and amidation reaction which was previously reported (Scheme 1) [25]. First, EDA and MA were individually dissolved in methanol, then the EDA solution was added dropwise into the MA solution (20% molar excess) to form odd-generation PAMAM. The even-generation PAMAM was similarly synthesized: the methanolic solution of odd-generation PAMAM was added dropwise into the solution EDA (100% molar excess). All reactions were kept in the dark for 2–8 days under nitrogen atmosphere at room temperature. The product PAMAM G3.0 was dialyzed in methanol for 3 days by membrane with MWCO = 3.5 kDa.

### 2.3. Synthesis of G3.0@mPEG

Before being conjugated with G3.0, mPEG was firstly activated with NPC. The mPEG was melted in a three-neck round bottom flask at 60 °C for 2 h under nitrogen flow. NPC was quickly added into completely melted mPEG, then the mixture was magnetically stirred for 6 h at 60 °C, dissolved in THF and kept for 6 h at room temperature. The activated mPEG was crystallized in cold diethyl ether, filtered by Buchner funnel, and dried by rotary evaporator.

The activated mPEG was conjugated with G3.0 via the reaction between terminal amine groups of G3.0 and carbonate groups of mPEG-NPC to form carbamate groups and release 4-nitrophenol. The G3.0 and mPEG-NPC were individually dissolved in deionized water (DW), after that the mPEG-NPC solution was added dropwise into the G3.0 solution. The reaction was kept in dark at room temperature for 24 h, then dialyzed by membrane with MWCO = 3.5 kDa in water for 3 days. Finally the dialyzed solution was lyophilized to yield purified G3.0@mPEG [26].

### 2.4. Characterization of G3.0@mPEG

The chemical structure of G3.0 and G3.0@mPEG were analyzed by ^1^H-NMR (Bruker AC 500 MHz spectrometer, Bruker Co., Billerica, MA, USA) and FT-IR (Bruker Equinox 55 FTIR spectrometer, Bruker, Ettlingen, Germany). The morphology of particles was characterized by TEM (JEM-1400, JEOL, Tokyo, Japan).

### 2.5. Quantitative Analysis of Drugs

The drugs were quantified by HPLC method with reversed-phase silica C18 column (150 × 4.6 mm, particle size 5 µm). Mobile phase was the mixture of phosphate buffer (pH = 2.5) and methanol with ratio 65:35 (*v*/*v*). The flow rate was 1.0 mL/min. The UV wavelength of detector for 3TC and AZT was 266 nm.

### 2.6. Encapsulation of Retrovirus Drugs

For 3TC and AZT, the drug solutions were prepared by dissolving drugs in purified water (2 mg/mL) and stirring for one hour. The drug solution was then added dropwise into G3.0@mPEG solution (8 mg/mL of water) until the ratio of drug and G3.0@mPEG was 1:4 *w*/*w*, stirred for 24 h at room temperature to form ARV@G3.0@mPEG. After that, the mixture was transferred into dialysis tube with MWCO = 3.5 kDa and dialyzed in 250 mL of purified water or SDS 0.1% solution for 30 min. The dialysate was exchanged three times, then the non-encapsulated drug in dialysate was quantified by HPLC to calculate the encapsulation efficiency (EE%). The solution inside the dialysis tube was lyophilized to obtain the dry ARV@G3.0@mPEG.

The EE% was calculated by the Equation (1):

(1) $$\mathrm{EE}\%=\frac{\mathrm{weight}\;\mathrm{of}\;\mathrm{loading}\;\mathrm{drug}}{\mathrm{weight}\;\mathrm{of}\;\mathrm{drug}\;\mathrm{in}\;\mathrm{feed}}\;\times \;100\%$$

### 2.7. Drug Release Experiments

Dialysis technique and HPLC were used to obtain the drugs release profile of ARV@G3.0@mPEG systems. In detail, 500 mg of dried ARV@G3.0@mPEG was dissolved in 10 mL of various buffer solutions with pH of 1.2, 4.5, 6.8 and 7.4, and transferred into dialysis tube. Subsequently, the dialysis tube was put into the silver paper-covered beaker containing 500 mL of buffer and magnetically stirred at 100 rpm. After the first hour, 1.0 mL of sample was collected and replaced by the same volume of fresh buffer solution every two hours.

### 2.8. Drug Release Kinetic Study

The in vitro release patterns of G3.0@mPEG loading 3TC and AZT were analyzed by two drug release kinetic models, including zero-order, first-order, Higuchi and Korsmeyer–Peppas. The zero-order kinetic model examined the relationship between time and cumulative percent drug release. The first-order kinetic model measured the relationship of between time and log cumulative percent of drug remaining. The Higuchi model assessed the relationship between the square root of time and cumulative percent drug release, and the Korsmeyer–Peppas measured the relationship between time and log cumulative percent drug release. Graphs of these models were created based on below corresponding equations using Microsoft Excel, and then the rate constant and correlation values were obtained by applying a linear regression fit [27,28].

The zero order model describes drug release which is concentration-independent (Equation (2)):

(2) $$C=k_{0}t$$

The first order model describes drug release, which is concentration-dependent (Equation (3)):

(3) log(100−C)=−kft2.303

The Higuchi model describes drug release from matrix and polymeric systems (Equation (4)):

(4) $$C=k_{H}\sqrt{\;t\;}$$

The Korsmeyer–Peppas model describes drug release from matrix and polymeric systems (Equation (5)):

(5) $$C=k_{K}t^{n}$$

where C is the cumulative% drug released at time t, *k*_0_ is the zero order rate constant, *k*_f_ is the first order rate constant, *k_H_* is the Higuchi constant, *k_K_* is the Korsmeyer–Peppas constant, and *n* is the exponent that describes a particular diffusion mechanism.

### 2.9. Cytotoxicity

The cytotoxicity was evaluated on human fibroblast cells, BJ cell line (ATCC^®^ CRL-2522^TM^) via MTT assay. The sample solutions with the concentration of 200,000 µg/mL in DMSO were prepared in Eppendorf tubes by vortex for 1 min at 2000 rpm and ultrasonication at 37 °C for 20 min. The solutions were then diluted to 1000 µg/mL before exposing to BJ cells.

The BJ cells were seeded in 96-well plate with 104 cells/well and incubated at 37 °C, relative humidity of 98% and CO_2_ of 5%. The cell culture media was the DMEM mixing with 10% FBS and 0.1% penicillin-streptomycin, which is shortly called DMEM. After 1 day, the cell media was replaced by sample solutions with various concentrations, such as 0, 5, 25, 50, 100, 200, 400 and 800 µg/mL for G3.0; and 0, 50, 100, 200, 400 and 800 µg/mL for G3.0@mPEG and ARV@G3.0@mPEG. The negative control sample was DMSO 0.5% dissolved in DMEM. Each sample was repeated three times for three wells. After 24 h incubating at 37 °C, relative humidity of 98% and CO_2_ of 5%, 1 µL of MTT (ab211091, Abcam) was added and the wells were continuously incubated in the same condition for 4 h. The media was then replaced by 200 µL of DMSO. After lightly shaking for 5 min, the cell viability was determined by measuring optical density at 570 nm using ELISA, HumanRead. The Prism software was used to determined IC50. Meanwhile, live/dead assay based on dual staining acridine orange (AO)/propidium iodide (PI) was used to evaluate the morphology of BJ cells with assistance of a dual laser channels confocal microscope.

### 2.10. Anti-HIV Effect

HIV-1 proteases is indispensable enzymes in the HIV life cycle and is considered one of the most important targets in anti-HIV drugs development [24,29]. Pepsin and HIV-1 are in the family of aspartic proteases with similar primary structure, the same activating mechanism and are inactivated by pepstatin A inhibitor. Therefore, pepsin can be used as an alternative target enzyme for HIV-1 protease inhibitory evaluation [23,24]. 

The anti-HIV activity of free ARV and loaded ARV was evaluated based on pepsin inhibitory activity using Enzymatic Assay of Pepsin (3.4.23.1) with hemoglobin as a substrate. It is a determination of spectrophotometric stop rate. The Enzymatic Assay of Pepsin was conducted at 37 °C and pH 2.0 as described in Table 1.

The pepsin inhibition activity of the samples was expressed as a percentage of remaining activity of pepsin in comparison to the test at 0 mM of drug (taken as 100%), using the following Equation (6):

(6) $$\mathrm{Remaining}\;\mathrm{activity}\;\mathrm{of}\;\mathrm{pepsin}\;\left(\%\right)=\frac{{\;A}_{\mathrm{Test}}\;{-\;A}_{\mathrm{blank}}\;\;}{A_{\mathrm{Test}\;0}\;{-\;A}_{\mathrm{blank}}}\times \;100\%$$

where: A_Test_: absorbance of tests at different drug concentrations from 0 to 0.35 mM. A_Test 0_: absorbance of the test at 0 mM of drug. A_blank_: absorbance of blank.

## 3. Results

### 3.1. Chemical Structure of G3.0@mPEG

**Figure 1:**
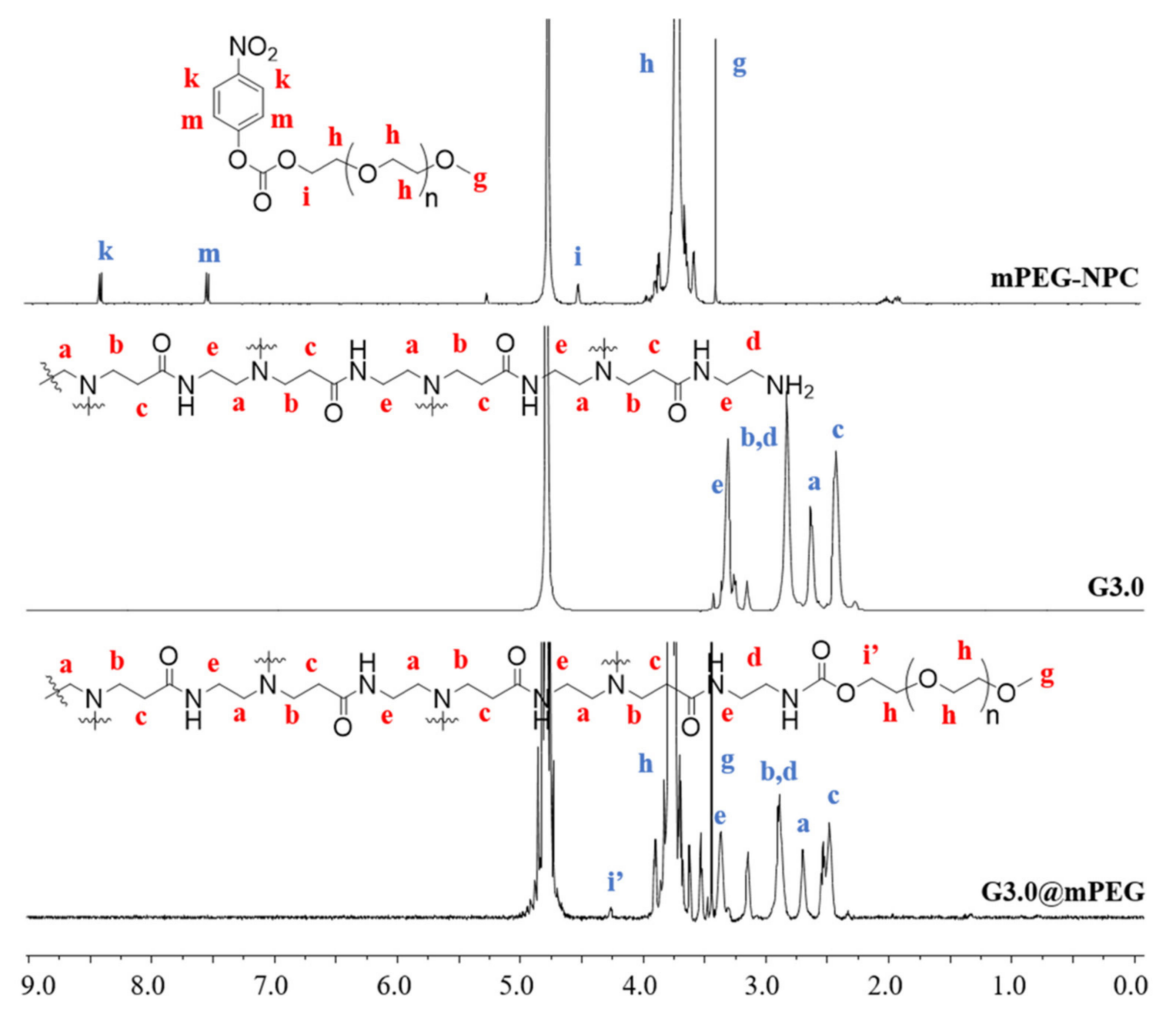
^1^H-NMR spectrum of G3.0@mPEG, G3.0 and mPEG-NPC. The lowercase Roman letters indicate protons with respective peaks.

**Figure 2:**
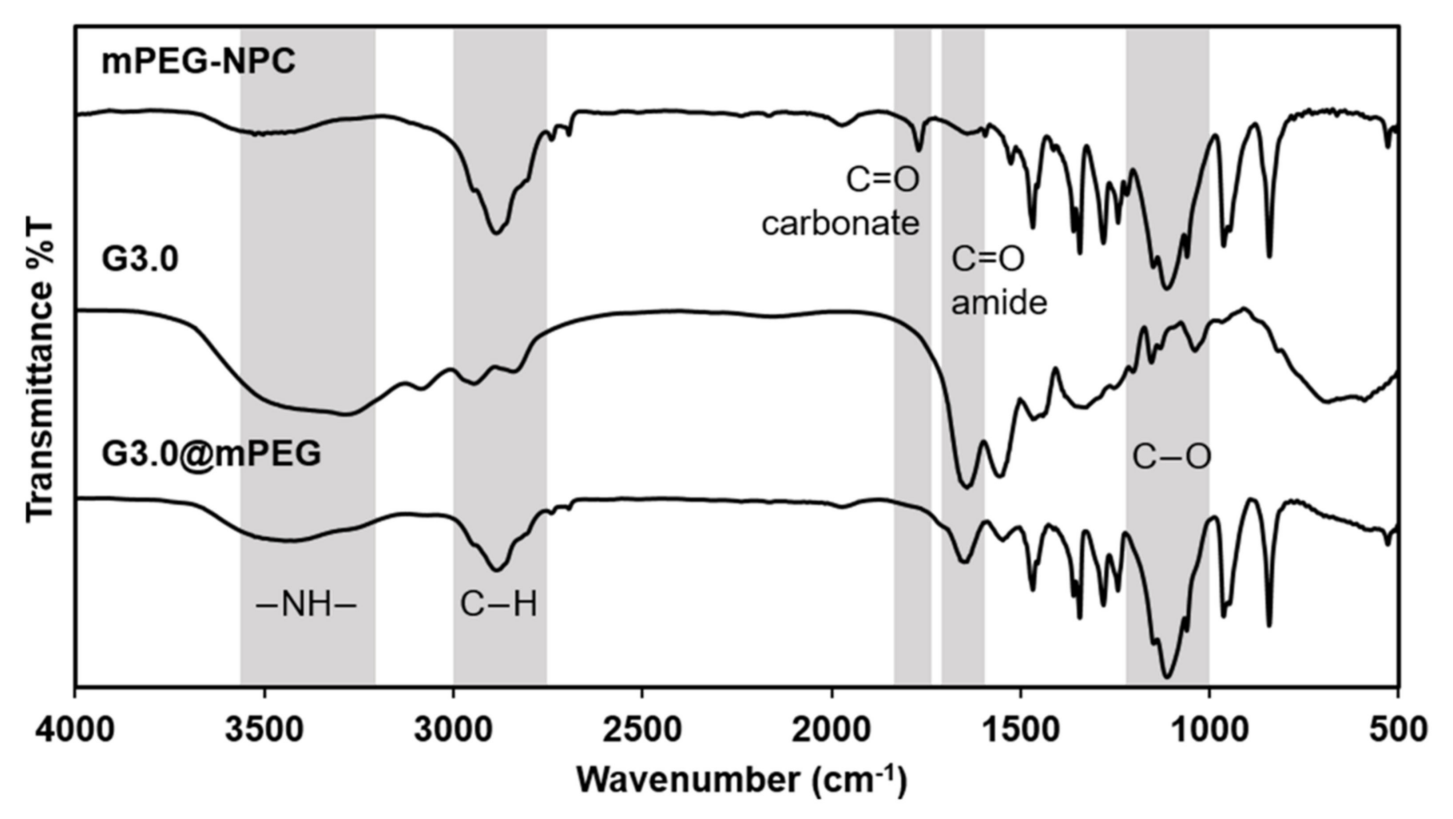
FT-IR spectra of mPEG-NPC, G3.0 and G3.0@mPEG.

The G3.0 was synthesized step by step via Michael reaction and amidation reaction. Subsequently, NPC-activated mPEG was conjugated on G3.0 surface to form G3.0@mPEG and release nitrophenol. The chemical structure of G3.0, mPEG-NPC, and G3.0@mPEG was characterized by ^1^H-NMR and FT-IR. The ^1^H-NMR and FT-IR results were shown in Figure 1 and Figure 2 below, respectively.

In the ^1^H-NMR spectra of G3.0, the protons at δH = 2.960–2.720 ppm (peak b, d) and δH = 2.720–2.550 ppm (peak a) were assigned to protons in methylene groups of -CH2-NH-. Meanwhile the proton at δH = 2.550–2.350 ppm (peak c) was attributed to protons in methylene groups of -CH2-C(=O)- and the proton at δH = 3.330 ppm (peak e) was assigned to protons in groups of -CH2-NH-C(=O)-. These signals demonstrated that G3.0 was successfully synthesized and characterized [30].

In the ^1^H NMR spectra of PEG-NPC, the signals at δH = 3.400 ppm (peak g), δH = 4.010–3.500 ppm (peak h) and δH = 4.517 ppm (peak i) were assigned to protons of PEG, whereas the signals at δH = 7.542–7.517 ppm (peak m) and δH = 8.402–8.383 ppm (peak k) were specific protons of NPC. The spectra indicated that PEG was successfully activated by NPC [31].

In the ^1^H NMR spectra of G3.0@mPEG, there are specific signals of PAMAM at δH = 2.610–2.390 ppm (peak c), δH = 2.780–2.610 ppm (peak a), δH = 2.980–2.780 ppm (peak b, d), δH = 3.420–3.270 ppm (peak e), and. The specific signals of PEG (peak g and h) are not significantly different from which of the spectra of PEG-NPC. However, the signals of NPC (peak m and k) were disappeared, and peak i shifted from δH = 4.517 ppm (on spectra of PEG-NPC) to δH = 4.265 ppm (on spectra of G3.0@mPEG). This was due to the change of group >CH-CH2-OC(O)O- (peak i) to group >CH-CH2-OC(O)NH- (peak i’). These signals demonstrate that PEG was conjugated with G3.0 [32,33,34].

Figure 2 showed the FT-IR spectrum of mPEG-NPC, G3.0 and G3.0@mPEG. In the spectra of mPEG-NPC, the peak at 2888 cm^−1^ was C-H stretching vibration, the peak at 1114 cm^−1^ was the C-O asymmetric stretch and the peak at 1768 cm^−1^ indicated vibration of C=O stretch of carbonate group [35,36]. In spectra of G3.0, the appearance of peak at 3415–3265 cm^−1^ was attributed to secondary amine group (-NH-). The strong peak at 1645 cm^−1^ was C=O stretch of amide group, and the one at 1556 cm^−1^ was N-H bending vibration. In the spectra of G3.0@mPEG, there were peaks of G3.0 at 3345–3356 cm^−1^ (N-H) and 1641 cm^−1^ (C=O of amide); and peak of PEG at 1112 cm^−1^ (C-O) [35,37]. Besides, the disappearance of peak at 1768 cm^−1^ was due to the replacement of carbonate group by carbamate group, which demonstrate that PEG was conjugated with G3.0 [36].

### 3.2. Morphology of PAMAM G3.0@mPEG 

**Figure 3:**
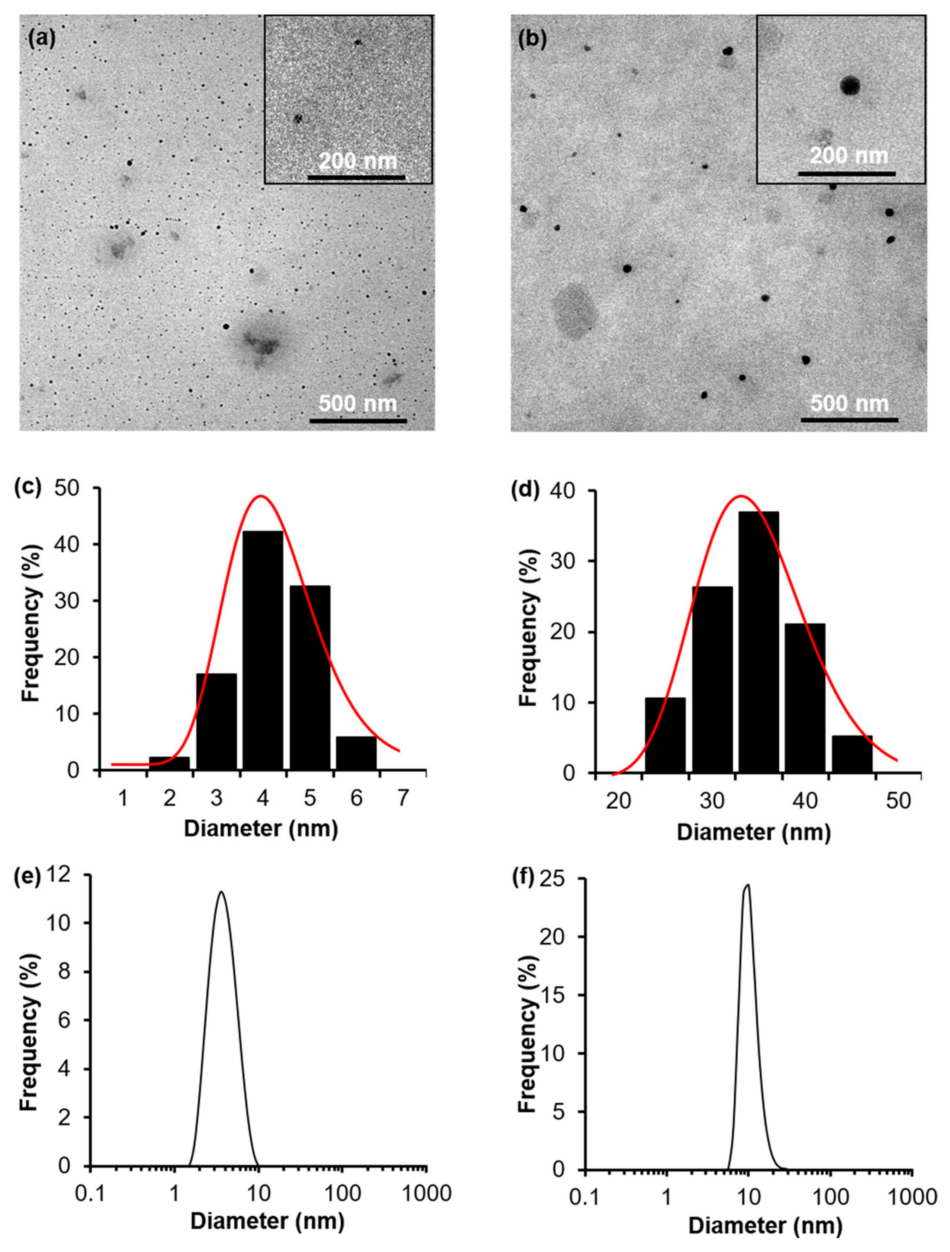
TEM (**a**,**b**), statistical graph from TEM (**c**,**d**) and DLS (**e**,**f**) results of G3.0 and G3.0@mPEG, respectively.

The morphology and particle size of dendrimer G3.0 and G3.0@mPEG was shown in Figure 3. Obviously, the TEM images (Figure 3a,b) showed that both G3.0 and G3.0@mPEG particles were sphere. The particle size from TEM images was measured using ImageJ software, then calculated and expressed as mean ± standard deviation, which were 4.3 ± 0.9 nm for G3.0 and 34.5 ± 4.6 nm for G3.0@mPEG. The comparison of the particle size between G3.0 and G3.0@mPEG using one-way ANOVA showed that the two particle sizes were statistically different with *p* < 0.005. Meanwhile, the hydrodynamic size of G3.0 and G3.0@mPEG were determined by dynamic light scattering technique (DLS) with 5.8 ± 0.6 nm for G3.0 and 123.5 ± 5.2 nm for G3.0@mPEG. Both TEM and DLS results of G3.0@mPEG were bigger than those of G3.0 due to the mPEG layer conjugating on G3.0 surface. This layer not only holds more drugs but also enhances the blood residence time of the particles [22]. However, the DLS results were bigger than the diameter measured by TEM, which was explained due to the solvate layer around the particle in DLS measurement [37].

### 3.3. Cytotoxicity

**Figure 4:**
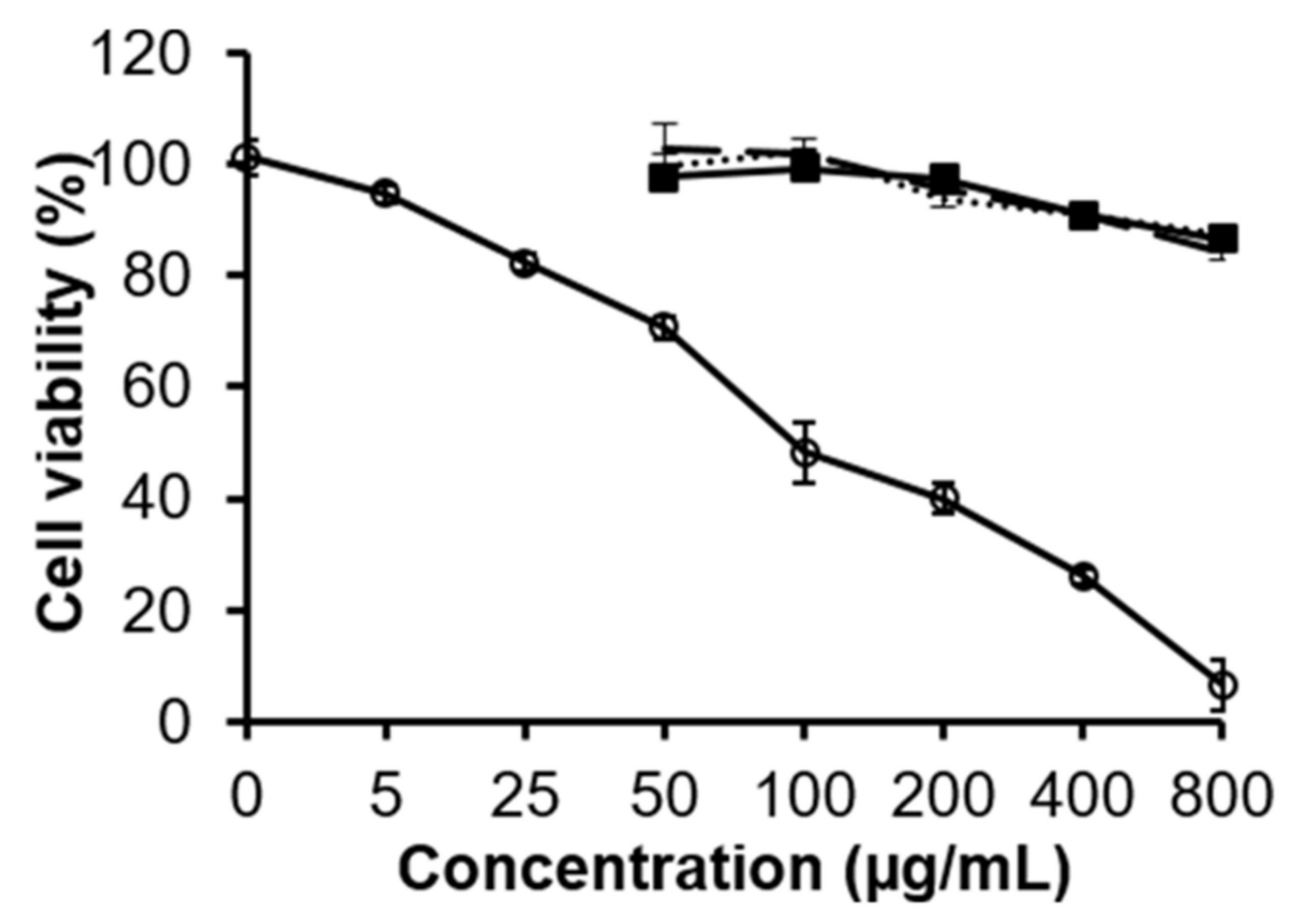
Viability of BJ cells that were exposed to G3.0 (empty circle, solid line), G3.0@mPEG (solid square, solid line), 3TC@G3.0@mPEG (round dot) and AZT@G3.0@mPEG (long dash) solutions within 24 h, calculated by using OD data (n = 3).

Along with the sustainable drug release, cytotoxicity of nano cargo is especially important. Therefore, toxicity of full formulation as well as non-drug loaded cargo were tested in this study. To evaluate the cytotoxicity of the carrier system, the G3.0, G3.0@mPEG and ARV@G3.0@mPEG were tested with human fibroblast BJ cells. Figure 4 showed that viability of cells was decreased from 100% to 6.6% when the concentration of G3.0 increased from 0 to 800 µg/mL, with the IC50 was 102.06 ± 6.4 µg/mL. The high cytotoxicity of G3.0 was due to the high positive charge (measured as 40.9 ± 2.1 mV) caused by amine groups on the surface of G3.0, which interacts with the negative-charged cells membrane [38]. When being surface modified with mPEG, a biocompatible polymer, the positive charge of G3.0@mPEG was reduced to 17.3 ± 1.9 mV, results in low cytotoxicity. Even when exposed G3.0@mPEG at the highest concentration in this experiment (800 µg/mL), more than 80% of cell still alive [22,39]. These results proved that G3.0@mPEG was a biocompatible delivery system that can be used in medical applications.

For 3TC and AZT-loaded G3.0@mPEG, cell viability was higher than 80% when the treated concentration up to 200 µg/mL. This demonstrated the low cytotoxicity of G3.0@mPEG loading drugs.

**Figure 5:**
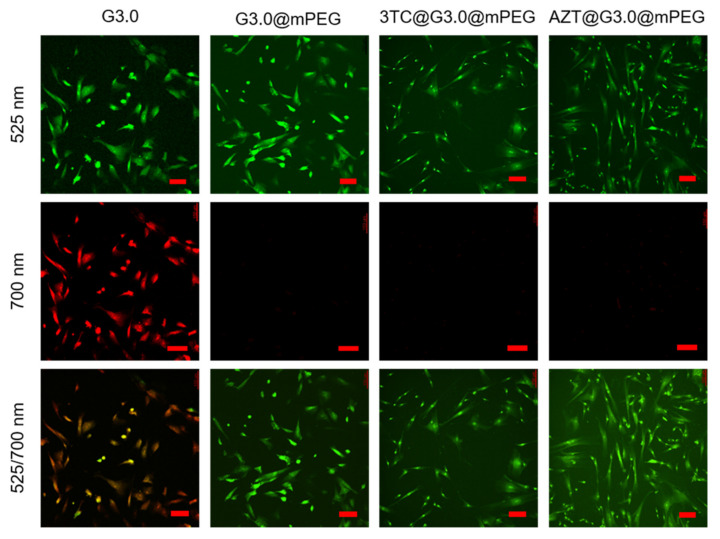
Images of AO/PI dual stained BJ cell line treated by G3.0, G3.0@mPEG, 3TC@G3.0@mPEG and AZT@G3.0@mPEG solutions at 200 µg/mL within 24 h, observed under 525 and 700 nm laser channels. The scale bar is 150 µm.

Figure 5 showed the images of AO/PI dual stained BJ cell line treated by G3.0, G3.0@mPEG, 3TC@G3.0@mPEG and AZT@G3.0@mPEG solutions at 200 µg/mL within 24 h. At the wavelength of 700 nm, G3.0@mPEG, 3TC@G3.0@mPEG and AZT@G3.0@mPEG performed no red fluorescence. This proved that the synthesized material G3.0@mPEG as well as the loading drug systems 3TC@G3.0@mPEG and AZT@G3.0@mPEG caused no cytotoxicity against BJ cells. In contrast, PI entered the damaged cell membrane caused by G3.0 and bind to the DNA, producing very strong red fluorescence.

### 3.4. Drug Encapsulation of PAMAM G3.0@mPEG

In this study, the two ARV drugs including 3TC and AZT were used to evaluate the drug loading ability and drug release kinetic of PAMAM G3.0@mPEG. The results shown in Table 2 indicate that the drug loading efficiency (DLE) and drug loading content (DLC) of 3TC and AZT loading systems were approximately 32% and 7%, respectively. The DLE and DLC of G3.0@mPEG for AZT and 3TC were equivalent. There was no significant difference possibly because AZT and 3TC were both water soluble drugs. Therefore, the retained drug of AZT and 3TC in the carrier could be due to the hydrogen and electrostatic bonds between the drugs and the carrier. Compare to another study of loading ARV into nanoparticles, G3.0@mPEG showed its significantly high ARV loading capacity [40].

### 3.5. Drug Release Experiments

**Figure 6:**
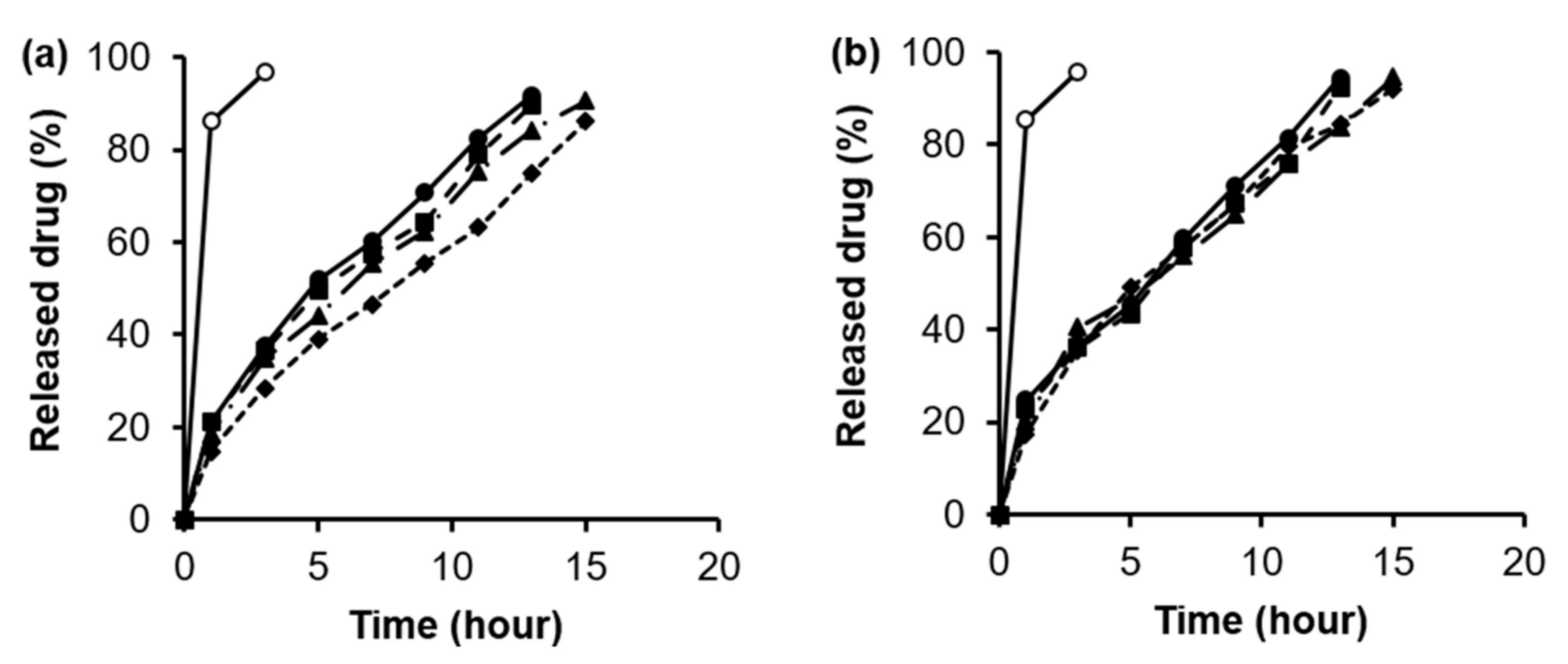
Release profile of free and loaded 3TC (**a**) and AZT (**b**) in 4 different pH conditions. Empty circle: free drugs at pH 7.4; Solid circle: loaded drugs at pH 1.2; Solid square: loaded drug at pH 4.5; Solid triangle: loaded drugs at pH 6.8; Solid diamond: loaded drugs at pH 7.4.

The drug release profile of two free ARV drugs and ARV@G3.0@mPEG systems in various pH were showed in Figure 6. The free 3TC and AZT were fast release within first three hours, as 96.68% and 95.84%, respectively. Meanwhile, the ARV@G3.0@mPEG exhibited a sustained release profile in both 4 pH conditions. In first three hours, the released 3TC and AZT were 3.4 and 2.7 fold lower than released free drugs. After 15 h, released 3TC was more than 80% of the initial amount (86.04%), while released AZT was slightly higher (92.04% of the initial amount).

The release pattern of the two ARV@G3.0@mPEG systems in media with pH = 7.4 from Figure 5 were fitted to four models, including zero-order, first-order, Higuchi and Korsmeyer–Peppas to determine the highest correlation with experimental results. It has been reported that the first 60% of drug release is typically sufficient for determining the best fit model for drug release [41]. Therefore, release data of 3TC at 9 h (55.51%) and AZT at 7 h (58.02%) were used to fit into the four models. The kinetic release parameters and regression coefficients calculated from the four kinetic models are shown in Table 3.

Both loaded 3TC and AZT showed the highest correlation with the Korsmeyer–Peppas model (*R*^2^ = 0.9997 and 0.9985, respectively). The slope value more than 0.45 (*n* > 0.6) was found with the Korsmeyer–Peppas’s equation for G3.0@mPEG loading these three drugs formulations, which corresponds to a non-Fickian transport [42].

### 3.6. Anti-HIV Effect

**Figure 7:**
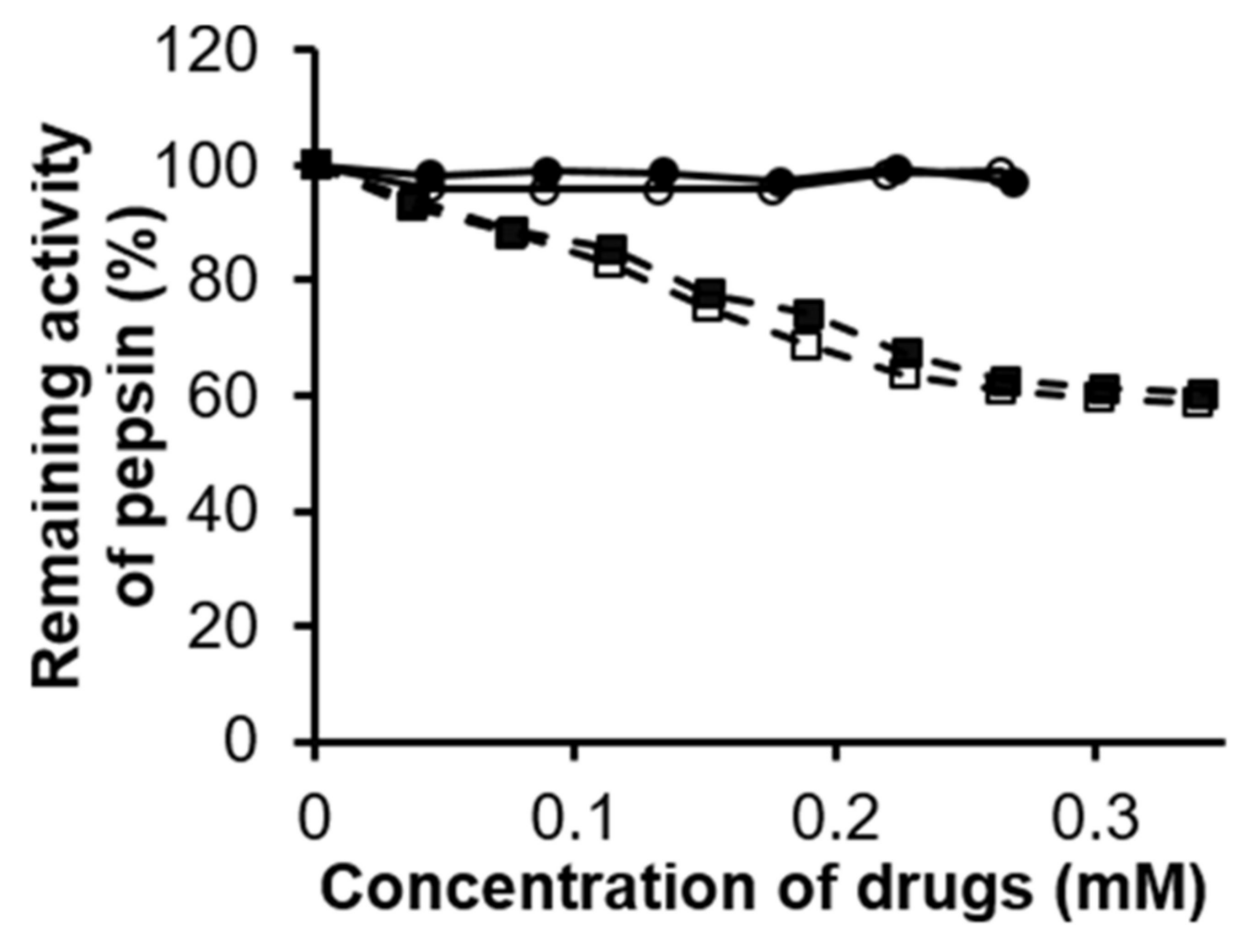
Pepsin inhibitory activity of free ARV and ARV@G3.0@mPEG. Empty circle: free 3TC, Solid circle: 3TC@G3.0@mPEG; Empty square: AZT, Solid square: AZT@G3.0@mPEG.

The anti-HIV activity of the free ARV and loaded ARV was evaluated through Enzymatic Assay of Pepsin. The result was presented in Figure 7. In general, the pepsin inhibition activities of each ARV remained stable after being loaded into G3.0@mPEG systems.

Both 3TC and AZT belong to the drug group called nucleoside and nucleotide analog reverse transcriptase inhibitors (NRTIs). However, their pepsin inhibition activities were different. At the concentrations of 0.264 mM for 3TC, the percentage of residual pepsin activity was 98.40%. Therefore, it could be said that 3TC had no pepsin inhibition activity. Meanwhile at a concentration of 0.038 mM for AZT, the percentage of remaining activity of pepsin was 92.95% (inhibition of about 7% of pepsin activity) and at a concentration of 0.226 mM AZT the remaining percentage of pepsin activity was 63.68% (36% inhibition of pepsin activity). This demonstrated that AZT had the ability to mildly inhibit pepsin. The results of Enzymatic Assay of Pepsin are consistent with the theoretical characteristics of NRTI group [43].

## 4. Conclusions

In this work, PAMAM G3.0 was successfully synthesized and surface modified with mPEG. Both PAMAM G3.0 and G3.0@mPEG particles have spherical morphology with a particle size of 4.3 ± 0.9 nm and 34.5 ± 4.6 nm, respectively. Both G3.0@mPEG and ARV@G3.0@mPEG showed no cytotoxicity on the human fibroblast BJ cells. The drug loading efficiency and drug loading content of G3.0@mPEG for individually 3TC and AZT were around 40% and 9%, respectively. G3.0@mPEG loading both the ARV performed sustained drug release behavior which was best fitted with the Korsmeyer–Peppas model. Importantly, the anti-HIV activity of ARV remained after being loaded in the G3.0@mPEG. The findings in this study demonstrate that PAMAM@mPEG is a promising nanocarrier system for loading ARV in HIV treatment and prevention.

## Figures and Tables

**Table 1:** Reagents and steps for Enzymatic Assay of Pepsin.

Reagents	Blank (mL)	Test (mL)
Hb Solution ^1^	5.0	5.0
Incubate at 37 °C for 10 min		
Enzyme Solution ^2^	-	1.0
Mix well, incubate at 37 °C for exactly 10 min		
TCA Solution ^3^	10.0	10.0
Enzyme Solution ^2^	1.0	-
Mix well, incubate at 37 °C for 5 min		
Filtrate through 0.45 µm syringe filter, measure absorbance at 280 nm		

^1^ Hb Solution: 2.0% (*w*/*v*) hemoglobin substrate solution in 10 mM hydrochloric acid. ^2^ Enzyme Solution: pepsin insoluble enzyme containing 15–25 units/mL and samples (free ARV and loaded ARV) at different drug concentrations from 0 to 0.35 mM in 10 mM hydrochloric acid solution. ^3^ TCA Solution: 5% (*w*/*v*) Trichloroacetic Acid Solution.

**Table 2:** DLE and DLC of G3.0-PEG loading ARV.

Drugs	m G3.0-PEG (mg)	m Drug (mg)	DLE (%)	DLC (%)
3TC	357.76	83.07	32.09	7.03
AZT	313.60	83.50	32.17	7.18

**Table 3:** Rate constants and correlation coefficients of ARV@G3.0@mPEG systems in pH = 7.4 condition obtained through some mathematic models.

System	Zero-Order		First-Order		Higuchi		Korsmeyer–Peppas	
	*k*_0_	*R* ^2^	*k_f_*	*R* ^2^	*k_H_*	*R^2^*	*n*	*R* ^2^
G3.0-PEG@3TC	5.807	0.958	0.037	0.990	18.49	0.993	0.601	0.999
G3.0-PEG@AZT	8.027	0.953	0.053	0.990	22.35	0.993	0.625	0.999

## Data Availability

All data generated or analyzed during this study are included in this published article.
